# Ex Vivo Overactivation of Lymphocyte Subsets in Fibrotic Hypersensitivity Pneumonitis Is Blunted by a Sphingosine-1-Phosphate Receptor Ligand

**DOI:** 10.3390/ijms26073197

**Published:** 2025-03-29

**Authors:** Olivier Courtemanche, Carole-Ann Huppé, Pascale Blais-Lecours, Cloé Maranda, Mathieu C. Morissette, Marie-Renée Blanchet, Geneviève Dion, David Marsolais

**Affiliations:** 1 Centre de recherche de l’Institut Universitaire de cardiologie et de pneumologie de Québec, 2725 Chemin Sainte-Foy, Quebec City, QC G1V 4G5, Canada; olivier.courtemanche.2@ulaval.ca (O.C.); carole-ann.huppe.1@ulaval.ca (C.-A.H.); pascale.blais-lecours@criucpq.ulaval.ca (P.B.-L.); cloe.maranda.1@ulaval.ca (C.M.); mathieu.morissette@criucpq.ulaval.ca (M.C.M.); marie-renee.blanchet@criucpq.ulaval.ca (M.-R.B.); genevieve.dion@criucpq.ulaval.ca (G.D.); 2 Department of Medicine, Faculty of Medicine, Université Laval, Quebec City, QC G1V 0A6, Canada

**Keywords:** extrinsic allergic alveolitis, BAFF, cytokine, TLR9, ozanimod, S1P, S1P_1_, S1P_5_, CD69, TNF

## Abstract

Lymphocytes are central to the pathogenesis of hypersensitivity pneumonitis and a strong body of evidence supports that lymphocytes are modulated by sphingosine-1-phosphate receptor-modifying drugs. This exploratory study aimed to determine if a pharmacological sphingosine-1-phosphate receptor ligand interfered with the activation of lymphocytes obtained from fibrotic hypersensitivity pneumonitis patients. Peripheral blood mononuclear cells of 12 patients and 10 control subjects were submitted to CD3/CD28 stimulation, isolated B cells were incubated with a TLR9 ligand; and we tested how these stimulations were impacted by ozanimod, a sphingosine-1-phosphate receptor ligand. T cell and B cell subsets from patients overexpressed CD69 and cytokines such as TNF and IL-4 in response to CD3/CD28 stimulation, compared to controls. In patients with fibrotic hypersensitivity pneumonitis, ozanimod alleviated CD3/CD28 induction of CD69, IL-4, and TNF in CD8, but not CD4 T cells. In isolated B cells stimulated with a TLR9 ligand, ozanimod reduced cell surface expression of CD69, CD86, and CD40, as well as TNF and IL-6 accumulation in supernatant. We conclude that lymphocyte subsets are functionally impacted in patients with fibrotic hypersensitivity pneumonitis and that ozanimod can interfere ex vivo with the overactivation of B cells and CD8 T cells in response to specific stimuli.

## 1. Introduction

Hypersensitivity pneumonitis (HP) is caused by recurrent exposure to airborne antigens found in bioaerosols [1]. So far, hundreds of agents have been shown to cause HP and deleterious bioaerosols feature both antigens susceptible to activate T cells and adjuvants such as Toll-like receptor (TLR) ligands, which could contribute to the disease’s pathogenesis [2,3]. Endogenous TLR9 ligands, such as free circulating mitochondrial DNA may also contribute to the pathogenesis of HP, as their airway and circulating concentrations are increased [4]. The incidence rate of the disease (1.28–1.94 cases per 100,000 person/year in the USA [5]) is likely underestimated since fibrotic HP (fHP) is often misdiagnosed as idiopathic pulmonary fibrosis [6].

Multiple pieces of evidence suggest that lymphocytes are central to interstitial lung diseases, including nonfibrotic HP and fHP [7,8,9], which remain difficult to manage using conventional pharmacological approaches [10]. We showed that co-modulation of T cells and B cells was required for optimal alleviation of experimental HP [11]. Case reports and retrospective studies suggest clinical improvement following last-resort anti-CD20 immunotherapy or with anti-metabolites in patients with HP [12,13]. Although immunotherapies emerge as promising avenues, reported improvements remain mild and partial, underscoring the need to further explore disease mechanisms along with novel pharmacological options.

S1P receptors, especially S1P_1_, are major regulators of T cell and B cell biology [14] and S1P receptor-targeting drugs emerge as effective options for chronic immune diseases [15]. For instance, S1P_1_ is critical for lymphocyte egress from specific lymphoid tissues and it counter-regulates CD69, which is an early activation marker responsible for tissue accumulation and homing of lymphocytes. Several studies made in rodents demonstrated that S1P_1_ was involved in the release of soluble mediators and may affect cell surface expression of stimulatory and co-stimulatory molecules. Indeed, S1P_1_ transgenic overexpression and S1P_1_ mutations that interfere with receptor internalization favor IL-17 production by CD4 T cells through the IL-6-JAK-STAT3 pathway [16,17]. Ligands targeting S1P_1_ and favoring its internalization also reduce TH1 and TH2-associated cytokine production upon TCR stimulation in a dose-dependent manner, and upon TLR activation with LPS [18,19].

In experimental HP, we showed that the levels of the S1P_1_ receptor were modulated on T cells and B cells at different stages of the model; and that an S1P_1_ chemical ligand inhibited pulmonary accumulation of antigen-specific antibodies [20]. We also provided evidence that an S1P receptor ligand directly prevented murine B cell activation induced by a TLR ligand [3]. Yet, whether S1P receptor ligands may modify lymphocyte functions in the context of human HP remains to be determined. In this exploratory study, we determined if a sphingosine-1-phosphate receptor ligand could directly interfere with the activation of T and B lymphocytes obtained from patients with fHP.

## 2. Results

### 2.1. Subject’s Characteristics

Ten control subjects (six men and four women) and twelve patients with fHP (seven men and five women) were recruited in each group, whose average age and smoking statuses were similar (Table 1). Six fHP patients were under immunosuppressive treatment at the time of the study and six out of the twelve fHP patients had been exposed to a known antigen. Characteristics of the participants are presented in Table 1. According to the published diagnostic criteria from the official clinical practice guidelines of ATS/JRS/ALAT 2020 [21], 8 out of the 12 participants met the criteria for a definitive diagnosis of hypersensitivity pneumonitis, 3 for a high-confidence diagnosis, and 1 for a low-confidence diagnosis. Regarding the participant with a low-confidence diagnosis, he was a farmer presenting with significant lymphocytosis in the bronchoalveolar lavage (55%) but an indeterminate pattern for HP on a high-resolution computed tomography scan based on the latest diagnostic criteria. A diagnosis of fHP was retained as the most likely diagnosis after an expert multidisciplinary discussion. The diagnosis was maintained until his death, after more than 25 years of follow-up.

### 2.2. Increased Circulating Factors in fHP Patients

Multiple cellular sources contribute to increased circulating cytokine levels under inflammatory conditions. Compared to control subjects, fHP patients had increased plasma levels of interleukin (IL)-21, tumor necrosis factor (TNF), and IL-4 and a tendency for increased IL-17A levels (Table 2). B cell activating factor (BAFF), a key factor in B cell mobilization and activation, was also increased in fHP patients (median [Q_1_–Q_3_]: 469.3 [419.5–564.5] pg/mL in control subjects vs. 593.7 [492.7–937.0] pg/mL in fHP patients). Similarly, plasma levels of the B cell chemoattractant C-X-C motif chemokine ligand (CXCL)13 were also elevated in fHP patients compared to control subjects (median [Q_1_–Q_3_]: 0.9 [0.0–19.5] pg/mL in control subjects vs. 59.7 [15.1–125.5] pg/mL in fHP patients) (Table 2). In contrast, the concentration of CXCL12 was similar between both groups (Table 2). Altogether, these results support that fHP patients feature increased inflammatory cytokine levels and increased activation/chemotactic factors for B cells in circulation.

### 2.3. Indications That Specific Lymphocyte Subsets Are Primed and/or Activated in fHP Patients

**Figure 1:**
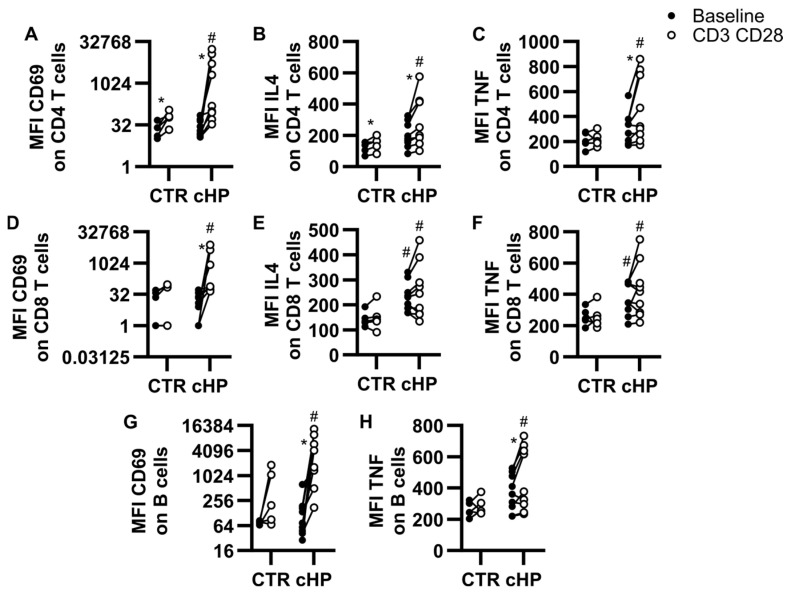
Activation profiles of lymphocyte subsets in PBMC cultures. Peripheral blood mononuclear cells (PBMC) from control (CTR) subjects or patients with fibrotic hypersensitivity pneumonitis (fHP) were cultured in the absence (baseline, black dots) or in the presence of anti-CD3/CD28-coupled microbeads (CD3 CD28, empty dots). Median fluorescence intensity (MFI) for CD69 (**A**,**D**,**G**), IL-4 (**B**,**E**), and TNF (**C**,**F**,**H**) was assessed on CD4 T cells (**A**–**C**), CD8 T cells (**D**–**F**) and B cells (**G**,**H**). Two-way ANOVA was used for control vs. fHP comparisons and # denotes a significant difference against homologous baseline or CD3/CD28 condition. Within groups, the effect of CD3/CD28 stimulation vs. baseline was assessed using paired bidirectional *t*-tests, and * denotes a significant difference. Dots represent individual values, which are connected with a black line for the same individual. * *p* < 0.05, n = 5 to 9 per group.

CD69 is an early activation marker. Peripheral blood mononuclear cells (PBMC) were incubated with anti-CD3/CD28-coupled microbeads to induce pan T cell activation or left untouched for baseline comparison (Figure 1). At baseline, CD69 median fluorescence intensity (MFI) did not differ between the control and fHP groups on both CD4 and CD8 T cells (Figure 1A,D). Within individual groups, CD3/28 stimulation increased cell-surface expression of CD69 on CD4 T cells from control subjects (Figure 1A) and on both CD4 and CD8 T cells from fHP patients (Figure 1A,D), compared to baseline. When comparing samples stimulated with anti-CD3/CD28-coupled microbeads, CD69 MFI was significantly increased in both CD4 and CD8 T cells of fHP patients, compared to the control group (Figure 1A,D).

Intracellular cytokine staining can be used to assess lymphocyte activation, priming, and polarity. When comparing to fluorescence minus one (FMO) controls, IL-4 and TNF were detectable in all samples tested (Supplementary Figure S1), while interferon (IFN)γ MFIs were very low or undetectable in the majority of patients. At baseline, MFI levels for IL-4 and TNF were higher in the fHP group than in the control group in CD8 (Figure 1E,F), but not CD4 T cells (Figure 1B,C). The CD3/CD28 stimulation significantly increased IL-4 and TNF MFI in CD4 T cells (Figure 1B,C) for both the control and fHP groups. However, this increase did not reach significance in CD8 T cells (Figure 1E,F; IL-4, *p* = 0.13; TNF, *p* = 0.14). Nevertheless, when comparing control and fHP groups under the CD3/CD28 stimulation condition, absolute MFI for IL-4 and TNF were higher in both CD4 and CD8 T cells of fHP patients compared to control subjects.

Noteworthy of mention, T cell-specific CD3/CD28 activation in PBMC also caused an indirect activation of B cells, which increased CD69 and TNF MFI, when compared to baseline (Figure 1G,H).

### 2.4. Ozanimod Inhibits CD8 T Cell Activation Ex Vivo

**Figure 2:**
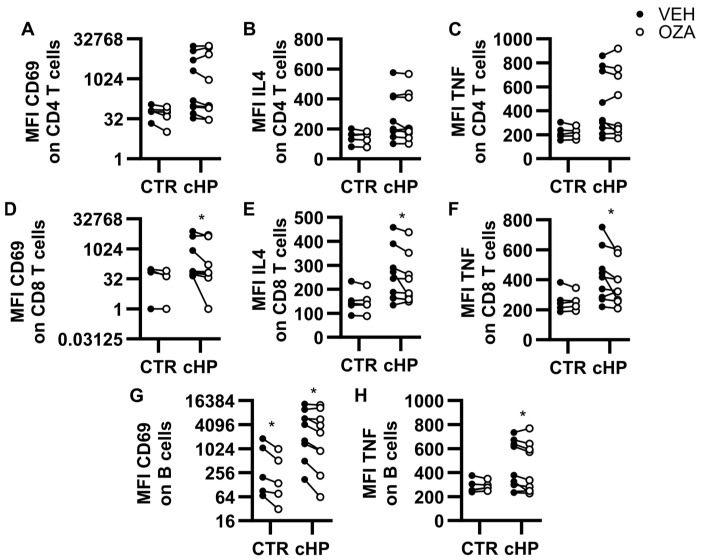
Ozanimod inhibits lymphocyte subset activation in PBMC cultures stimulated with anti-CD3/CD28-coupled microbeads. Peripheral blood mononuclear cells (PBMC) from the control group (CTR) or patients with fibrotic hypersensitivity pneumonitis (fHP) incubated with anti-CD3/CD28-coupled microbeads in the presence of a vehicle (VEH, black dots) or ozanimod (1 µM, empty dots). Median fluorescence intensity (MFI) for CD69 (**A**,**D**,**G**), IL-4 (**B**,**E**), and TNF (**C**,**F**,**H**) were assessed on CD4 T cells (**A**–**C**), CD8 T cells (**D**–**F**), and B cells (**G**,**H**). The effect of ozanimod vs. vehicle was assessed using paired bidirectional *t*-tests and * denotes a significant difference. Dots represent individual values, which are connected with a black line for the same individual. * *p* < 0.05, n = 5 to 9 per group.

PBMCs were incubated with ozanimod (1 µM) or with the vehicle for 1 h before adding anti-CD3/CD28-coupled microbeads (Figure 2). Twenty-four hours later, cell surface levels of CD69 and intracellular levels of IL-4 and TNF were compared. For both control and fHP groups, ozanimod did not impact CD69 (Figure 2A), IL-4 (Figure 2B), or TNF (Figure 2C) MFIs in CD4 T cells, compared to vehicle. In CD8 T cells, ozanimod did not impact the surface expression of CD69 (Figure 2D) or MFIs for IL-4 (Figure 2E) and TNF (Figure 2F) in the control group. However, in fHP patients, CD69, IL-4, and TNF levels were all decreased in CD8 T cells, compared to vehicle, when incubated with ozanimod. We also analyzed B cells, since they were activated in PBMC cultures submitted to the CD3/CD28 stimulation. Under these conditions, ozanimod reduced CD69 B cell surface levels in both control and fHP groups, compared to the vehicle (Figure 2G). A mild inhibitory effect of ozanimod was also observed for TNF in B cells, which only reached statistical significance in the fHP group (Figure 2H).

### 2.5. Ozanimod Perturbs B Cell Activation in Response to a TLR9 Ligand

**Figure 3:**
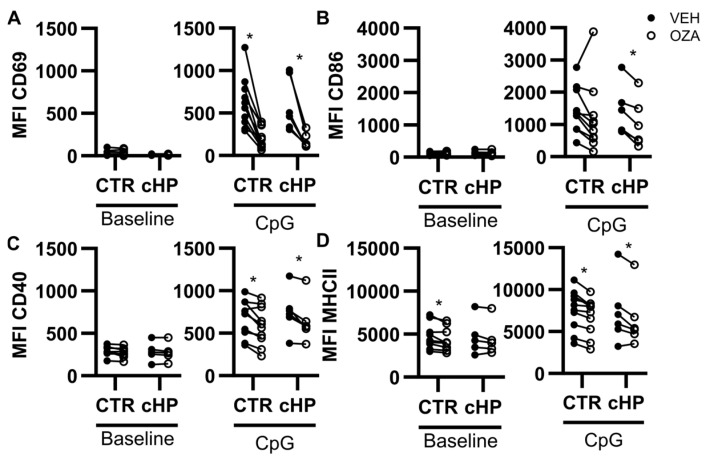
Ozanimod inhibits CpG-induced B cell activation in isolated B cell cultures. Isolated B cells from individual control subjects (CTR) or fibrotic hypersensitivity pneumonitis (fHP) patients were cultured for 24 h with medium only (Baseline) or CpG ODN2006 3 μg/mL (CpG) in the absence (VEH, black dots) or presence of ozanimod (1 µM; empty dots), Median fluorescence intensity (MFI) of CD69 (**A**), CD86 (**B**), CD40 (**C**), and MHCII (**D**) was assessed. n = 5 to 10 per group. Paired bidirectional *t*-tests were performed for VEH vs. ozanimod comparisons. * *p* < 0.05.

We recently showed that an S1P_1_ receptor ligand prevented murine B cell activation induced by a TLR ligand [3]. Since human B cells express TLR9, we focused on the impact of ozanimod in response to the TLR9 ligand CpG. Under non-stimulated conditions (baseline), MFI for CD69 and CD86 were low, while CD40 and Major Histocompatibility Complex II (MHCII) were easily detectable in all individuals with no difference between groups (Figure 3). B cells from control subjects and fHP patients were responsive to CpG, which increased cell surface expression of CD69, CD86, CD40, and MHCII (Figure 3). Under baseline conditions, ozanimod mildly decreased MHCII surface expression on B cells from the control group (Figure 3D). However, ozanimod potently inhibited CpG-induced upregulation of CD69 and CD86, especially in fHP patients. Its effect on CpG-induced increases in CD40 (Figure 3C) and MHCII (Figure 3D) was moderate, but significant in both groups.

**Figure 4:**
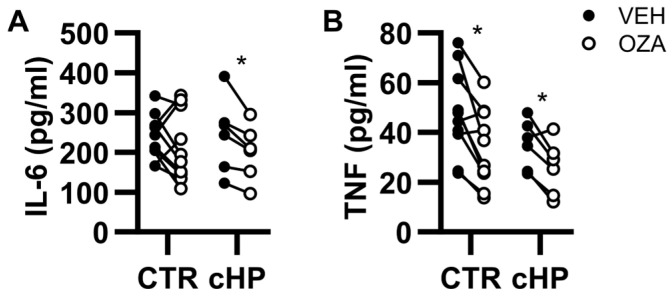
Ozanimod reduces CpG-induced TNF and IL-6 supernatant accumulation in isolated B cell cultures. Isolated B cells from individual control (CTR) subjects or fibrotic hypersensitivity pneumonitis (fHP) patients were incubated with CpG ODN2006 (3 μg/mL) in the absence (black dots) or presence of ozanimod (1 µM; empty dots) for 24 h. The concentration of IL-6 (**A**) and TNF (**B**) in the cell-free supernatant was quantified using ELISA. In total, 6 to 10 individuals were included for each experimental condition based on the availability of B cells. Paired bidirectional *t*-tests were performed for VEH vs. ozanimod comparisons. * p < 0.05.

At baseline, the concentrations of TNF and IL-6 in B cell culture supernatant were below the limits of detection and CpG caused the accumulation of TNF and IL-6 for all individuals in both control and fHP groups (Figure 4). Compared to vehicle, ozanimod reduced supernatant levels of IL-6 in fHP patients only (18% inhibition); and of TNF in both control subjects (28% inhibition) and fHP patients (26% inhibition) (Figure 4A,B).

## 3. Discussion

In this exploratory study, we first determined that patients had dysregulated levels of circulating factors consistent with mixed inflammation and fibrotic presentations of interstitial lung diseases. We also found that, in cultured PBMCs, T cells displayed signs of being primed and/or overactivated in the fHP group compared to the control group. According to our analyses, we did not find isolated B cell cultures from fHP patients to be activated at baseline, nor did they appear to respond more strongly than control B cell cultures to CpG ex vivo. Importantly, ozanimod dampened the activation of T cells in PBMC cultures incubated with a T cell receptor activation mimic. Noteworthy of mention, under these experimental conditions, paracrine activation of B cells was also inhibited by ozanimod. When B cells were isolated, ozanimod directly yet mildly inhibited CpG-induced B cell activation and cytokine release. This study supports the concept that lymphocyte alterations are prominent in fHP patients and unveils sphingolipid-modifying drugs as potential therapeutic tools impacting directly with lymphocyte functions, or with their interplay, in order to dampen their activation.

In line with HP models [22] and with prior observations made in humans [23], we found that several inflammatory mediators were increased in the fHP group. Indeed, circulating IL-21 and likely IL-17A were elevated in our fHP group, aligning with the notion that a TH17 response contributes to HP pathogenesis [22]. Although the notion of a shift towards a TH2 response in fHP was challenged by murine studies [24], our current observation of elevated IL-4 in several fHP patients argues that such a paradigm could contribute to fibrotic remodeling. In line with this concept, we also detected an increase in BAFF, a B cell survival factor that has repeatedly been associated with interstitial lung diseases, particularly ones with a fibrotic component [25]. Altogether, the circulating mediator analysis is consistent with the notion that patients recruited in this study had hallmark features of fHP and were distinctive from their control counterparts.

Several studies documented alterations in PBMC components in the context of HP including transcriptomic modifications [8], modifications of T cell subpopulations [26], and the identification of dysregulated CD4 and CD8 T cells [27]. The current work addressed the concept that lymphocyte subsets could be functionally impacted in fHP patients in response to specific stimuli that we find relevant to fHP. Similar to published findings [9,28] we determined that some markers, in this case CD69, did not differ between the fHP and control groups at baseline. However, CD69 surface expression was greatly increased on both CD4 and CD8 T cells upon CD3/CD28 stimulation in fHP patients compared to the control group. This was also true for IL-4 and TNF in both CD4 and CD8 T cells, which is reminiscent of observations made in patients with interstitial lung diseases where a T cell receptor stimulation mimic increased the release of IL-2 from CD4 T cells [9], although no difference was documented for CD8 T cells in that study. Therefore, we suggest that the use of a stimulus that engages T cell receptor signaling cascades should be considered to further our understanding of functional alterations of CD4 and CD8 T cells in the specific context of HP.

Notwithstanding the early evidence that B cells play a crucial role in HP pathogenesis [29,30] in the lung, we found no published evidence arguing for major circulating B cell phenotypical alterations in nonfibrotic HP or fHP in humans. This aligns with single-cell RNA sequencing which revealed strong T cell leads in the context of HP, while no obvious B cell dysregulations were detected [27]. Here, CpG stimulation did not appear to elicit a particular phenotype in fHP patients compared to the control group in the isolated B cell culture model, but fundamental findings indicate that co-modulation of T cells and B cells is required to provide maximal reduction in inflammation experimental HP [11]. The current study may provide evidence for such a phenomenon in humans since we found that B cells from fHP patients feature higher levels of CD69 and TNF compared to the control group in PBMC cultures subjected to T cell-specific CD3/CD28 stimulation. Therefore, although B cells do not appear intrinsically activated or primed in fHP, they may contribute to amplifying the inflammatory milieu upon immunological stimuli involving T cells.

In line with the notion that lymphocytes are key players in HP pathogenesis, immune modulators are emerging as potential therapeutic options. S1P receptor modulators are relatively new drugs that notoriously alter T cell and B cell biology as well as the inflammatory response [14]. Several studies have addressed the concept that both CD4 and CD8 T cells are dysregulated and likely involved in the pathogenesis of HP, which agrees with our current findings. In a previous study, we also determined that an S1P receptor ligand interfered with inflammation and lymphocyte-related functions in a subchronic HP model [20]. In the current study, we show that ozanimod may impact the T cell receptor stimulation mimic-induced activation of CD8 T cells while not affecting CD4 T cells. While this result is difficult to explain, it should be noted that the pattern of expression of S1P_1_ differs on lymphocyte subsets in the course of experimental HP pathogenesis [3]. Moreover, although S1P_1_ is the main receptor expressed by lymphocytes, S1P_5_ was shown to be expressed on lymphocyte subsets under specific conditions [31]. Given that ozanimod is also active on S1P_5_, this may contribute to explaining the differing responses between lymphocyte subsets.

Our results also argue that S1P receptor modulation could counteract the upregulation of co-stimulatory molecules and the cytokine-mediated amplification of inflammation by B cells in fHP patients. It should be noted that, in addition to inhaled components, several endogenous TLR9 activators are increased in fHP patients, including mitochondrial DNA [4]. Although we have employed a synthetic TLR9 ligand, our results raise the possibility that in fHP, an endogenous TLR9 activation tone sustains deleterious functions of B cells. We thus speculate that S1P receptor pharmacological modulation could contribute to counteracting this phenomenon.

There is currently no cure for chronic fHP and pharmacological approaches aim to slow disease progression, reduce symptoms, and improve quality of life. These approaches include steroids and anti-metabolites whose primary mechanism of action is the reduction in lymphocytes, and anti-fibrotic agents such as nintedanib and pirfenidone. Beneficial effects of these drugs may also derive from their ability to reduce lymphocyte activation, and/or cytokine production [32,33,34]. The primary immunomodulatory mechanism of S1P receptor ligands is the sequestration of lymphocytes in secondary lymphoid organs [35]. Here, we show that an S1P receptor ligand, whose mechanistic target is not shared by currently used drugs, can also directly impact lymphocyte activation ex vivo.

This study has limitations, including its small sample size. Indeed, fHP is a heterogeneous disease, due among other things to the many genetic and environmental factors and co-morbidities often present in fHP patients [21,36], limiting the ability to detect subtle immunological changes. The use of primary samples is also a major limitation for studying S1P receptor dynamics and dysregulations, which has previously been carried out using cutting-edge genetic tools and murine models [37,38,39]. It should also be noted that this study features a single ozanimod dose design. However, CD69 modulation is a hallmark effect of S1P receptor-modulating drugs. The fact that ozanimod-induced effects were always accompanied by CD69 modulation suggests that S1P receptor modulation likely influences lymphocyte responses in fHP, and so, despite its complex pathophysiology.

In conclusion, we determined that a CD3/CD28 stimulation in PBMC ex vivo elicited functional differences in CD4 and CD8 T cells in fHP patients compared to a control group; and that ozanimod interfered with CD8 T cell activation. TLR9 activation of isolated B cell cultures induced the upregulation of CD69, CD86, and CD40, which was alleviated by ozanimod. In addition, ozanimod reduced the CpG-induced release of IL-6 and TNF in B cells of fHP patients ex vivo. Altogether, our results support the concept that T cells and their interplay with B cells are instrumental to fHP pathogenesis and unravel S1P receptor pharmacological modulators as putative agents to interfere with potentially deleterious lymphocyte functions in the context of fHP.

## 4. Materials and Methods

**Patients.** In total, 12 fHP patients and 10 healthy subjects with similar age, sex, and smoking status were recruited at the Institut Universitaire de Cardiologie et de Pneumologie de Québec (IUCPQ) between April 2019 and March 2021. A confident HP diagnosis was obtained following a multidisciplinary discussion and the confrontation of the clinical context, high-resolution computed tomography (HRCT), bronchoalveolar lavage (BAL) lymphocytosis, and lung biopsy (when available) using the Official ATS/JRS/ALAT Clinical Practice Guideline [21].

**PBMC Culture and CD3/CD28 Activation.** Venous blood was withdrawn during a single visit to the hospital and collected in K3EDTA-coated tubes. PBMC were isolated using Ficoll-Paque PLUS density gradient medium (GE Healthcare, Chicago, IL, USA). PBMC were incubated for 1 h at 37 °C to remove adherent monocytes. Plasma was centrifuged at 12,000× *g* for 15 min to remove platelets and stored at −80 °C. PBMC from healthy volunteers and chronic HP patients were plated with 500,000 cells per well in ImmunoCult™-XF T Cell Expansion Medium (StemCell, Vancouver, BC, Canada) supplemented with 10% FBS (Wisent, Saint-Jean-Baptiste, QC, Canada), 100 U/mL Penicillin/streptomycin (Wisent, Saint-Jean-Baptiste, QC, Canada), and 30 U/mL IL-2 (PeproTech, Cranbury, NJ, USA). PBMCs were pretreated with vehicle or 1 μM ozanimod (Medkoo, Durham, NC, USA) for 1 h before stimulation with 5 µL Dynabeads™ Human T-Activator CD3/CD28 for T Cell Expansion and Activation (ThermoFisher, Waltham, MA, USA) during 24 h.

**B cells culture conditions.** B cells were isolated by negative selection using EasySep Human B Cell Isolation Kit (StemCell, Vancouver, BC, Canada) and plated to a density of 100,000 cells/well in U-shaped 96 wells plates in complete culture medium (RPMI-1640 (Wisent, Saint-Jean-Baptiste, QC, Canada), 10% FBS and 100 U/mL Penicillin/streptomycin). B cells were left in a complete culture medium (baseline) or incubated for 24 h with 3 μg/mL CpG ODN 2006 (InvivoGen, San Diego, CA, USA). Baseline and CpG-stimulated B cells were co-incubated with a vehicle (medium) or with 1 μM ozanimod (Medkoo, Durham, NC, USA) for 24 h. The 1 µM ozanimod concentration was chosen based on previous studies that have used this dose for cell culture [40]. This concentration has been commonly employed to ensure sufficient receptor engagement and allow for the observation of downstream effects in cultured cells.

**Flow cytometry.** Cultured PBMC and cultured B cells were stained for flow cytometry analyses of sub-populations and activation markers for 20 min at 4 °C in the dark. Cells were then fixed in 1% paraformaldehyde for 1 h. For intracellular staining, cells were stained for 20 min in True-Nuclear™ perm buffer (BioLegend, San Diego, CA, USA). Antibodies are listed in Supplementary Table S1. Data were acquired using a FACS Diva-driven customized LSR Fortessa (BD Biosciences, Franklin Lake, NJ, USA), and the FlowJo software (Tree Star, Ashland, OR, USA, Version 10.8.1) was used for data analysis. FMO controls were used to set gates and to remove baseline signals to compute MFI.

**Soluble mediator quantification.** The plasmatic concentration of IL-17A, IL-6, IL-21, IL-22, TNF, IL-4, IFNγ, CXCL12, CXCL13, and BAFF were measured using DuoSet ELISA (R&D System, Minneapolis, MN, USA) according to the manufacturer’s instructions. 

**Statistical analyses.** The number of subjects/patients included in each experiment may vary depending on the availability of frozen samples. The number of individuals included in analyses is indicated for each figure. When the normality of data and assumption of homogeneity of variance were confirmed, data were presented as averages ± SEM. Individual data points were also shown. Two-way ANOVA or paired bidirectional *t*-tests were performed, when appropriate. The Mann–Whitney rank comparison test was used for data not fulfilling requirements for parametric analyses and the medians (Q1–Q3) are presented for each group. The significance threshold was set at *p* < 0.05.

## Figures and Tables

**Table 1:** Patients characteristics.

	Control Subjects (10)	Patients with fHP (12)
**General characteristics**		
Age (Year ± SEM)	65.3 ± 2.8	67.1 ± 7.1
Sex (Male, n *)	6	7
Smoker	0	0
Non-smoker	8	6
Former smoker	2	6
**fHP treatment at time of blood sampling**	N/D	
Mycophenolate and corticosteroids		5
Corticosteroids		1
Without fHP treatment		6
**Clinical data for diagnosis**		
Environmental exposure confirmed	N/D	6
HRCT **	N/D	
Typical fHP		9
Compatible for fHP		1
Indeterminate for fHP		2
Lymphocytosis	N/D	
Mild (10–20%)		1
Moderate (20–40%)		5
Important (>40%)		3
Biopsy **	N/D	10
Definite fHP		6
Probable fHP		4
Diagnosis confidence ** (Multidisciplinary team meeting)	N/D	
Definite fHP		8
High Confidence fHP		3
Low Confidence fHP		1

* Otherwise indicated, value designates the number of subjects; N/D: not determined. ** According to the ATS/JRS/ALAT Clinical Practice Guideline [21] and a multidisciplinary discussion.

**Table 2:** Plasma concentrations of cytokines.

Cytokine	Control (pg/mL) (n = 10)	fHP (pg/mL) (n = 9)	*p*-Value
TNF	0.0 (0.0–1.2)	10.7 (0.4–53.3)	0.020 *
CXCL13	0.9 (0.0–19.5)	59.7 (15.1–125.5	0.028 *
BAFF	469.3 (419.5–564.5)	593.7 (492.7–937.0)	0.035 *
IL-4	0.0 (0.0–0.6)	1.7 (0.1–11.9)	0.039 *
IL-21	0.0 (0.0–4.8)	29.3 (0.0–101.3)	0.050 *
IL-17A	0.0 (0.0–1.3)	1.7 (0.0–74.5)	0.089
IL-6	0.0 (0.0–7.6)	2.7 (0.0–33.4)	0.154
IL-22	110.0 (84.7–180.2)	212.2 (95.9–331.4)	0.278
CXCL12	99.9 (85.8–111.2)	105.7 (63.43–122.4)	0.720
IFNγ	1.0 (0.0–2.1)	1.2 (0.0–4.7)	0.923

Mann–Whitney *U* test. Median (Q_1_–Q_3_). Values are in pg/mL. * *p*-values under or equal to 0.05. n = 9–10.

## Data Availability

The anonymized data presented in this study are available upon request to the corresponding author.

## Supplementary Materials

The following supporting information can be downloaded at: https://www.mdpi.com/article/10.3390/ijms26073197/s1.
