# Supplementary material for: Ex Vivo Overactivation of Lymphocyte Subsets in Fibrotic Hypersensitivity Pneumonitis Is Blunted by a Sphingosine-1-Phosphate Receptor Ligand
Source: Int J Mol Sci. 2025 Mar 29;26(7):3197. doi: 10.3390/ijms26073197 (PMC11989070; doi:10.3390/ijms26073197)
Supplement: Supplementary file 1 [file ijms-26-03197-s001.zip › ijms-3524700-supplementary.pdf]

SUPPLEMENTARY MATERIAL

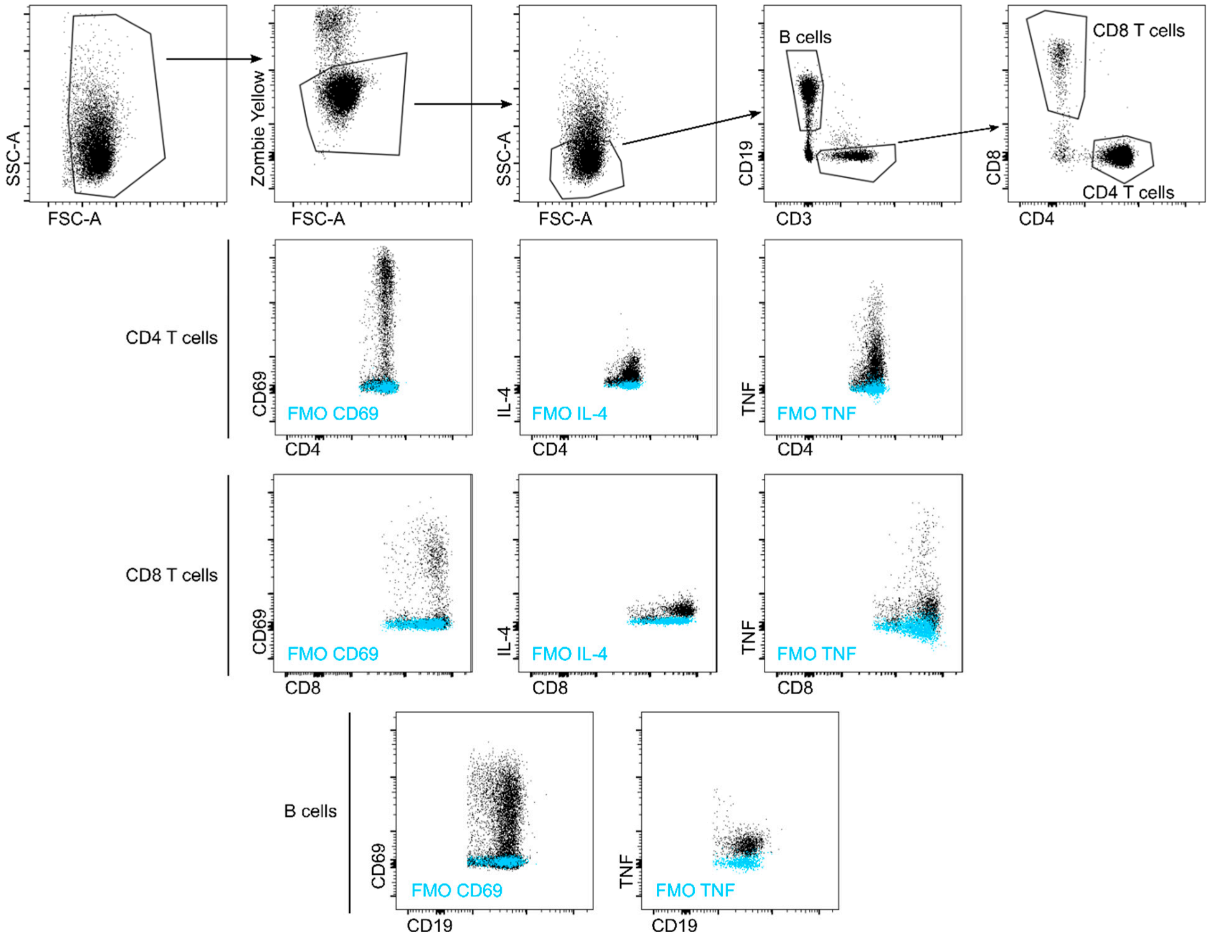

Supplementary Figure S1: Flow cytometry gating for CD3/CD28-stimulated PBMCs. PBMCs were isolated and incubated with anti-CD3/CD28-coupled microbeads for 24h and then processed for flow cytometry analyses. **Top row panels:** SSC-A^{low} FSC-A^{low} cells were selected from the Zombie Yellow^{low} gate. Then, B cells were gated as CD19^{+}CD3^{-}. From the CD19^{-}CD3^{+} gate, T cells were segregated as CD8^{+}CD4^{-} (CD8 T cells) and CD8^{-}CD4^{+} (CD4 T cells). **Second and third row panels:** CD69, IL-4 and TNF were analyzed in CD4 and CD8 T cells. **Bottom row:** CD69 and TNF were analyzed in B cells. Events (individual dots) from fully labeled samples are depicted in black, while fluorescence minus one (FMO) controls are shown in blue. Representative samples are shown.

Supplementary Table S1: Antibodies used for flow cytometry analyses

Antibody	Clone	Company
HLA-DR- PB	L243	BioLegend
CD40- PerCP	HI40a	Life Span Biosciences
CD69- PE	FN50	BioLegend
CD80- APC	2D10	BioLegend
CD86- APC Cy7	UCHL1	BioLegend
CD3- AF700	UCHT1	BD Bioscience
CD19- BV711	HIB19	BD Bioscience
CD4- PB	RPA-T4	BD Bioscience
CD8- BV786	HIT8α	BD Bioscience
TNF- PE-Cy7	Mab11	BD Bioscience
IL-4- FITC	MP4-25D2	BioLegend
Zombie Yellow		BioLegend
